# Robotic Resection of an Idiopathic Azygos Vein Aneurysm and the Diagnostic Role of Thoracic Venogram

**DOI:** 10.1177/15569845251334129

**Published:** 2025-05-16

**Authors:** Kayla M. Keenan, Rekha A. Cherian, Frank C. Lynch, Pauline H. Go

**Affiliations:** 1 The Pennsylvania State University College of Medicine, Hershey, PA, USA; 2 Department of Radiology, Penn State Milton S. Hershey Medical Center, PA, USA; 3 Division of Thoracic Surgery, Department of Surgery, Penn State Milton S. Hershey Medical Center, PA, USA

## Introduction

Azygos vein aneurysms (AVAs) are rare clinical entities, which are often asymptomatic and discovered incidentally on imaging.^1,2^ Contrast-enhanced computed tomography (CT) and magnetic resonance imaging (MRI) are commonly used, but poor lesion enhancement can lead to a misdiagnosis.^1,2^ Currently, there is no general consensus on the optimal diagnostic approach and treatment of asymptomatic, idiopathic AVAs. Both conservative and surgical approaches have been reported with favorable outcomes.^1,2^ In this case report, we highlight the utility of a multidisciplinary team approach to accurately diagnose an AVA in an otherwise healthy patient and describe a robotic approach to resection (Supplemental Video).

## Case Report

**Fig. 1.**
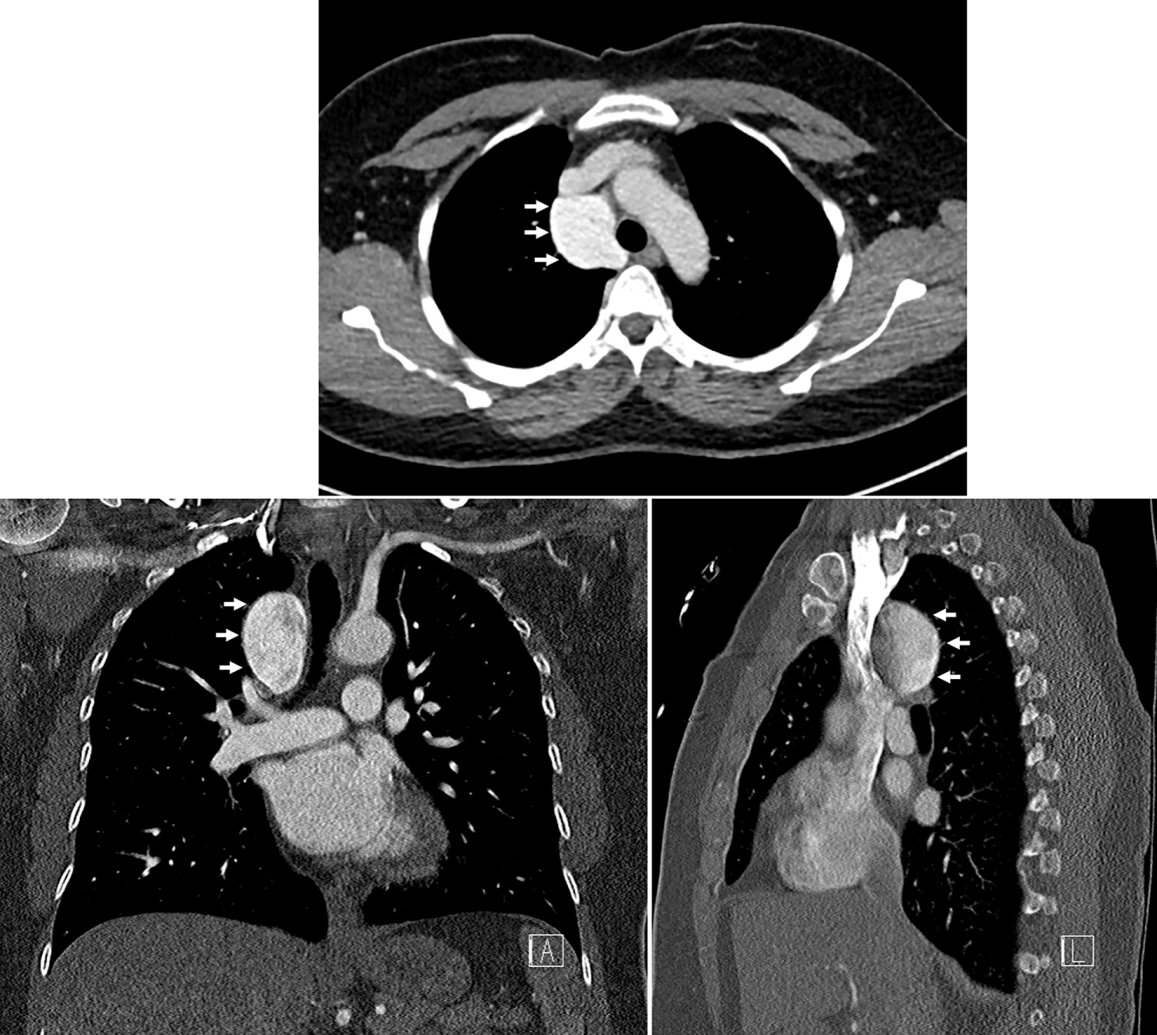
Chest computed tomography demonstrating a 4.4 × 4.1 × 3.6 cm mediastinal lesion adjacent to the superior vena cava and ascending aorta (white arrows), exhibiting contrast density with the appearance of flow but without direct communication to other vascular structures.

**Fig. 2.**
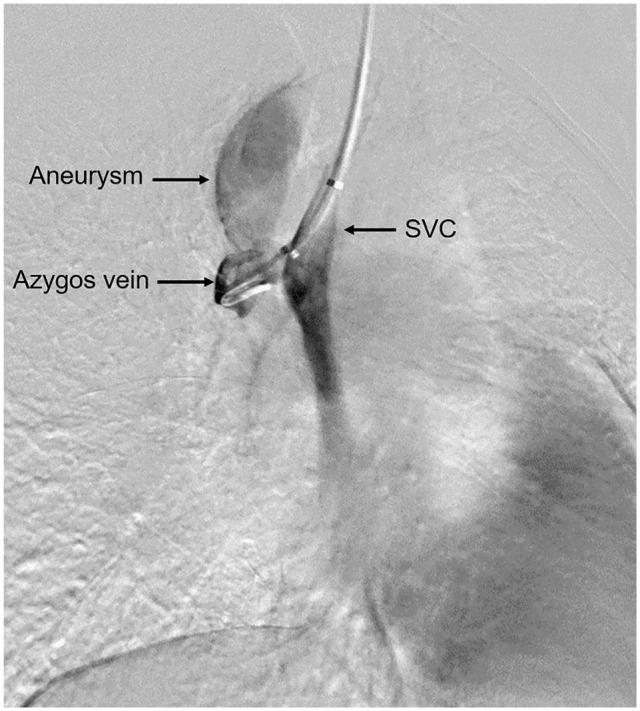
Thoracic venogram demonstrating a wide-mouthed venous aneurysm arising from the apex of the azygos arch and extending to its origin with the SVC, consistent with saccular-type azygos vein aneurysm. SVC, superior vena cava.

A 48-year-old healthy woman presented to her local emergency department following a motor vehicle collision, where she was the restrained driver hit at a high rate of speed by another vehicle. The patient reported significant left-sided head, shoulder, and flank pain. Vital signs and physical examination were unremarkable. Electrocardiogram showed no acute ST-segment deviations or repolarization abnormalities. Chest CT revealed a 4.4 × 4.1 × 3.6 cm mediastinal lesion adjacent to the superior vena cava (SVC) exhibiting intense contrast enhancement with the appearance of flow and no direct communication to other vascular structures (Fig. 1). This was reported as concerning for traumatic vascular malformation or injury. MRI showed a T2 hyperintense and intrinsically T1 isointense intensely enhancing lesion that remained suspicious for traumatic vascular malformation. Given ongoing concern for a vascular injury, she was transferred to our level 1 trauma center for further evaluation. Discussion between thoracic surgery and radiology led to repeat CT with delayed phase imaging, which showed a soft-tissue density lesion with persistent retention of contrast and slow filling. There was no continuity with adjacent vascular structures nor evidence of acute trauma in the region, suggesting a chronic venous malformation. In addition, review of a chest X-ray performed 18 months prior confirmed the presence of this lesion, making acute injury unlikely. Further collaboration with our interventional radiology group led to a thoracic venogram, demonstrating a wide-mouthed venous aneurysm arising from the apex of the azygos arch, extending to its origin with the SVC, confirming an AVA (Fig. 2). Its saccular morphology and proximity of the aneurysm neck to the origin precluded endovascular options for closure. Multiple options for management were presented to the patient, including a conservative watchful waiting approach based on clinical symptoms, active surveillance with serial imaging, or surgical resection. Due to significant anxiety over potential complications that can arise from an untreated AVA, the patient opted to proceed with resection.

General anesthesia through a double-lumen endotracheal tube was used. Robotic ports were placed along the seventh intercostal space. A large aneurysm originating from the azygos arch was identified abutting the SVC medially. Proximal control of the azygos vein was achieved between the aneurysm and SVC. Bleeding from attenuated portions of the aneurysm wall during dissection was easily controlled with focal pressure. The vein was divided proximally and distally with vascular staple loads. With the aneurysm in vascular discontinuity, it was dissected free and removed from the paratracheal space. A 24-French silicone drain was left in the chest. The patient’s postoperative course was uneventful. The chest drain was removed the following day, and she was discharged home later that day. Histopathology demonstrated findings consistent with aneurysm.

## Discussion

AVAs are rare causes of mediastinal mass seen on imaging and described only in isolated case reports and small cases series.^1^ Morphologically, they are classified into fusiform type, characterized by circumferential dilation of the vein, and saccular type, which are localized dilations within a portion of the vein wall.^2^ Idiopathic AVAs are typically asymptomatic and incidentally detected through routine medical imaging or unrelated procedures.^1^ When symptomatic, chest pain, dyspnea secondary to thrombosis, pulmonary embolism,^1^ tracheobronchial compression,^3^ dysphagia,^4^ and neurologic deficits^5^ have been reported. Their characteristic formation on the azygos arch is postulated to arise from a congenital weak point, supporting the belief that these represent congenital anomalies.^2^ Acquired AVAs, in contrast, are thought to result from pressure or volume overload states such as cardiac failure, portal hypertension, pregnancy, compression of the SVC due to malignancy, thrombus formation, or trauma.^1,2^

Contrast-enhanced CT and MRI are commonly used noninvasive diagnostic tools for AVAs. The presence of venous enhancement can facilitate the diagnosis of AVA, but frequently, poor contrast enhancement can lead to the misdiagnosis of a solid tumor or mediastinal lymphadenopathy.^1,4^ In these cases, a short-interval repeat CT scan using a larger volume of contrast with an additional 15 s delay can provide good venous enhancement and confirm the diagnosis of an AVA.^1^ In patients with dysphagia and dyspnea, the use of gastroscopy and fiber-optic bronchoscopy has also been reported.^1,4^ In our case, a definitive diagnosis remained elusive despite the use of standard imaging modalities and techniques. Only after collaboration with our radiology colleagues was the decision made to pursue more invasive imaging to establish an accurate diagnosis, allowing us to proceed with surgical resection in a safe and controlled manner.

To date, 73 cases of idiopathic AVAs have been reported worldwide,^4^ with the largest case series comprising 10 patients.^1^ With so few reported cases, no standard diagnostic or management guidelines exist to address asymptomatic AVAs when identified. Both surgical resection and conservative observation have been reported with good outcomes.^1^ Some authors advocate for early surgical resection to prevent complications such as rupture, thrombosis, and pulmonary thromboembolism.^2,6^ Although rupture has never been reported for AVAs, symptomatic pulmonary emboli instigated by large thrombosed AVAs were described by Ko et al.^1^ In this series, 7 of 10 AVAs were identified incidentally, and only 4 patients ultimately underwent resection. All others remained under surveillance without the development of any AVA-related symptoms or complications during the follow-up period. Kurihara et al. described a patient who formed an AVA thrombus during a 6-year follow-up period. She underwent an uncomplicated surgical resection with no adverse long-term sequelae.^6^

Surgical resection is recommended for symptomatic or enlarging AVAs.^1,6^ Multiple approaches to resection, including thoracotomy,^3,5–7^ and standard^8^ and single-port video-assisted thoracoscopy^4^ have been reported with good success and relatively quick recovery, with hospital discharge typically occurring within 2 to 4 days.^4,6,7^ Conversely, the management of asymptomatic AVAs is less clear. Patients should be counseled on the potential complications that can occur with untreated AVAs, and a plan for surveillance should be established for those choosing a nonoperative approach. Some have considered starting systemic anticoagulation to prevent thrombosis or pulmonary thromboembolism in these patients,^6^ but the risks of doing so must be heavily weighed against any potential benefits. In our case, the decision to resect an asymptomatic AVA was a shared one, driven primarily by the patient’s preference to alleviate ongoing anxiety. The robotic approach to resection permitted superior visualization of dissection planes and facilitated precise control of bleeding, enabling a rapid, uneventful recovery and discharge on the first postoperative day.

Our case highlights the importance of a multidisciplinary approach to the diagnosis and management of an idiopathic AVA and the role of thoracic venogram in cases in which standard imaging techniques are insufficient to provide definitive characterization. In circumstances where resection is indicated or desired, a robotic approach offers superior visualization of dissection planes, precise hemostasis, and faster recovery with earlier hospital discharge.
